# Physiological and transcriptome changes induced by *Pseudomonas putida* acquisition of an integrative and conjugative element

**DOI:** 10.1038/s41598-018-23858-6

**Published:** 2018-04-03

**Authors:** Ryo Miyazaki, Hirokazu Yano, Vladimir Sentchilo, Jan Roelof van der Meer

**Affiliations:** 1 Bioproduction Research Institute, National Institute of Advanced Industrial Science and Technology, Tsukuba, 305-8566 Japan; 2 Faculty of Life and Environmental Sciences, University of Tsukuba, Tsukuba, 305-8577 Japan; 3 Department of Fundamental Microbiology, University of Lausanne, Lausanne, 1015 Switzerland; 4 Present Address: Graduate School of Life Sciences, Tohoku University, Sendai, Japan

## Abstract

Integrative and conjugative elements (ICEs) comprise ubiquitous large mobile regions in prokaryotic chromosomes that transmit vertically to daughter cells and transfer horizontally to distantly related lineages. Their evolutionary success originates in maximized combined ICE-host fitness trade-offs, but how the ICE impacts on the host metabolism and physiology is poorly understood. Here we investigate global changes in the host genetic network and physiology of *Pseudomonas putida* with or without an integrated ICE*clc*, a model ICE widely distributed in proteobacterial genomes. Genome-wide gene expression differences were analyzed by RNA-seq using exponentially growing or stationary phase-restimulated cultures on 3-chlorobenzoate, an aromatic compound metabolizable thanks to specific ICE*clc*-located genes. We found that the presence of ICE*clc* imposes a variety of changes in global pathways such as cell cycle and amino acid metabolism, which were more numerous in stationary-restimulated than exponential phase cells. Unexpectedly, ICE*clc* stimulates cellular motility and leads to more rapid growth on 3-chlorobenzoate than cells carrying only the integrated *clc* genes. ICE*clc* also concomitantly activates the *P. putida* Pspu28-prophage, but this in itself did not provoke measurable fitness effects. ICE*clc* thus interferes in a number of cellular pathways, inducing both direct benefits as well as indirect costs in *P. putida*.

## Introduction

Horizontal gene transfer (HGT) is a major driving force for microbial evolution and adaptation, since transmission of genes from a donor bacterium can rapidly confer additional cellular functions and phenotypes to a naive recipient^1,2^. Typical ecological consequences of HGT are the emergence of bacterial species “suddenly” expressing antibiotic resistance^3–5^, new virulence factors^6,7^, or chemical degradation pathways^8–10^. HGT is frequently, but not exclusively, mediated by mobile DNA vectors, that are permissive for hitchhiking of auxiliary genetic material within their own boundaries. Mobile DNA vectors that promote HGT include, for example, plasmids, integrative and conjugative elements (ICEs) or bacteriophages^11^.

While acquisition of such auxiliary genes and their relevant phenotypes can be beneficial for hosts to adapt in particular ecological niches, it is generally assumed that the DNA acquisition also imposes various types of fitness costs on the cells. For example, horizontally transmitted genes may come with functional promoters that are active in the host, or intruded transferred regulatory genes may cross-talk to existing host global regulatory networks, resulting in perturbation of the genetic regulatory systems and subsequent fitness loss of the host^12^. Additional physiological and energetic costs may arise from increased need for replication of newly acquired DNA, transcription and translation of the acquired genes^13,14^, or cytotoxic effects of misfolded foreign proteins^15,16^. Costs inflicted by mobile vectors are in principle disadvantageous not only for the host but also for inheritance of the horizontally transmitted genes themselves, if we consider fitness of a mobile vector as maximizing its copy numbers in a given ecological niche. Hence, a variety of systems have evolved that minimize fitness costs of mobile DNAs. Several of these have been described in conjugative plasmids and temperate phages. For example, some conjugative plasmids encode “stealth” proteins to silence derogative functions that may impede plasmid maintenance^17–19^. Temperate phages express regulatory proteins, such as phage lambda’s cI repressor, that not only stably maintain the lysogenic state but also control host metabolic pathways to ensure host survival and efficient reproduction of phages^20–22^. In contrast to plasmids and phages, not much is known about the impact of ICEs upon acquisition by a new host.

ICEs are peculiar in that they have two life-styles. In the vertical transmission mode, they reside in the host chromosome^23,24^. Their segregation to daughter cells is guaranteed by chromosomal replication, like for lysogenic phages, instead of by independent replication, like for plasmids. In the horizontal transmission mode, the ICE excises from the chromosome through site-specific recombination and subsequently circularizes for conjugation, similar as in conjugative plasmid transfer^23,24^. In the recipient cell, the ICE molecule integrates site-specifically into the chromosome and is again stably maintained. Horizontal transmission of ICEs is proportionally rare, in the order of between 1 per 10^2^–10^8^ donor cells^25^. ICEs are widespread mobile elements, occurring in several evolutionary distinct families. They can carry ‘cargo’ genes encoding distinct functions, such as antibiotic resistance, heavy-metal resistance, symbiosis, or aromatic compound metabolism, from which the host can benefit under selective conditions^24,25^.

Here we address the question of measuring the global impact of an ICE in a new bacterial host. The model we use for our study is the ICE*clc* element, representative of a prevalent ICE-type among proteobacterial genomes^26^. ICE*clc* has a size of 103 kb and carries the *clc* genes, which provide the host with the capacity to degrade 3-chlorobenzoate (3CBA). It can transfer at a relatively high rate (3 per 10^2^ donor cells) from its original host *Pseudomonas knackmussii* B13 when the host has grown on 3CBA as a sole carbon source^27^. Transfer is initiated from specialized transfer-competent cells^28^, which arise during stationary phase as a result of a bistable expression mechanism^25^ and can conjugate the ICE when presented with new nutrients^29^. We have previously analyzed the global impact of ICE*clc* on its non-native host *Pseudomonas aeruginosa* PAO1. That study indicated less than an estimated 1% fitness loss and only few deviating individual host gene functions compared to PAO1 without ICE^30^. However, since ICE*clc* horizontal transmission from PAO1 as donor was very low compared to *P. knackmussii* (one per 10^5^ donor cells), and since that particular study could not address the impact of the ICE during growth on 3CBA, it may not have captured all relevant ICE-induced effects.

In order to complement previous data and obtain a wider picture of ICE*clc*-induced effects in a new host, we used here *Pseudomonas putida*. ICE*clc* transfer rates from *P. putida* as host are similar as *P. knackmussii* B13, and expression of key ICE*clc* promoters is non-distinguishable between both hosts, confined to a small (3–5%) bistable subpopulation of cells^28,31^. In order to study the impact of ICE*clc* during growth on 3CBA, we constructed a *P. putida* derivative without the ICE but with the *clc*-genes on the chromosome. We then analyzed genome-wide transcriptome differences in *P. putida* strains without or with ICE*clc* by using rRNA-depleted reverse-transcribed RNA sequencing. Cultures were grown on 3CBA as selective carbon source to induce highest ICE*clc* gene expression. We examined cells both from exponentially–growing conditions (hereafter, EXP phase) and from stationary phase, restimulated for 4.5 hours with fresh 3CBA (regrowth, or REG phase), under which the frequency of ICE*clc* horizontal transfer is maximal (3–5% of donor cells)^29^. Identified expression differences were subsequently profiled and substantiated by growth kinetics and physiology experiments. Finally, we verified cross-activation of a prophage in *P. putida* by ICE*clc* through single cell gene reporter experiments.

## Results

### Presence of ICE*clc* influences *P. putida* transcriptome differently depending on growth phase

Genome-wide transcription in *P. putida* with or without ICE*clc* was assessed from reverse-transcribed ribosomal-RNA depleted RNA samples, which were sequenced at on average 270-fold coverage by Illumina HiSeq and mapped to the *P. putida* genome (Table S1). Four replicates of the four different experimental treatments (two strains each in EXP or REG phase) clustered more closely together in multidimensional scaling analysis than between treatments, indicating clearly distinguishable global transcriptomes (Fig. S1).

**Figure 1:**
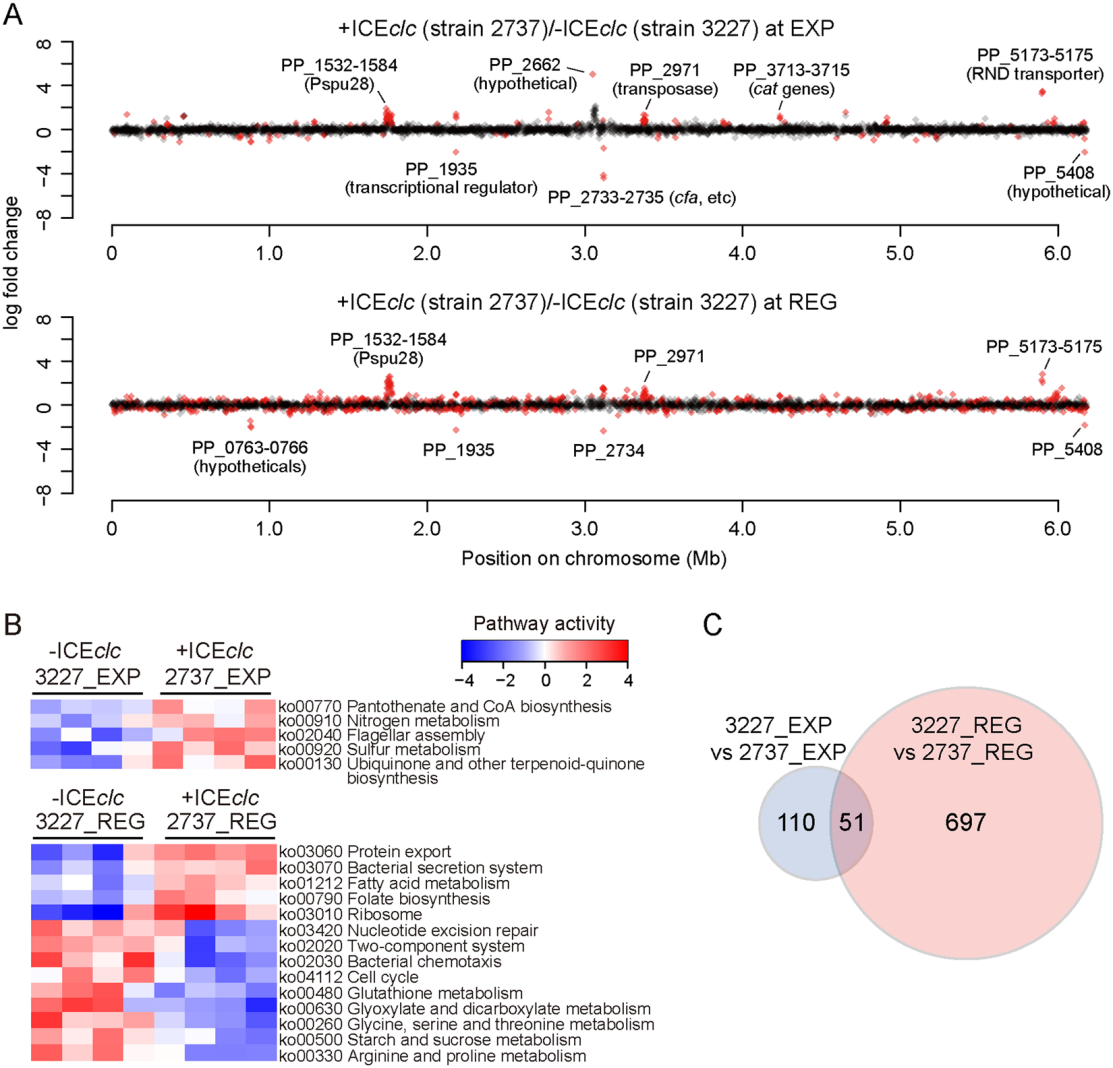
Gene expression profiles in presence or absence of ICE*clc*. (**A**) Log2–fold change of gene expression between *P. putida* with ICE*clc* (strain 2737) and that with *clc* genes only (strain 3227) in exponential (upper) and REG phase (lower). Only genes with an a.value (mean expression values, calculated using the trimmed mean of M values (TMM) normalization method) >4.0 are plotted, according to their positions on the chromosome. Differentially expressed genes with a q-value < 0.05 are coloured in red. (**B**) Pathway activity in the presence/absence of ICE*clc*. Activities were inferred from significantly induced or repressed KEGG Pathways identified for each dataset comparison (p < 0.05 in two-tailed Welch t test). Blue/red blocks indicate pathways (rows) that are up/down regulated in quadruplicate samples (columns) of specific strains and growth phases (top). Pathways are clustered based on the similarity of their activities across samples. (**C**) Venn diagram showing overlap among differentially expressed genes (DEGs) among transcriptome datasets. The intersection shows the DEGs at a q-value < 0.05 that were common to both comparisons and with the same direction of expression change.

In comparison to *P. putida* without ICE*clc* but with the *clc* genes (strain 3227), a total of 161 genes (outside ICE*clc* itself) were differentially expressed in *P. putida-*ICE*clc* (strain 2737) in EXP phase on 3CBA (FDR with q < 0.05; Fig. 1A, Data S1). Increased transcription of genes encoding RND-family transporter proteins (PP_3425–3427, 5173–5175), several types of transposases (PP_2964, 2971, 2974, 5405-5406), and a series of cryptic prophage Pspu28-related proteins (PP_1532–1584) was noticeable, whereas genes for extracellular-family sigma factors (PP_0352, 0704, 2192, 4208) were markedly lower expressed in strain 2737. Five KEGG–attributable pathways^32^, including nitrogen (ko00910) and sulfur metabolism (ko00920), flagellar assembly (ko02040), and biosynthesis of co-factors (ko00770, ko00130) were significantly higher expressed in *P. putida-*ICE*clc* than in *P. putida* without ICE in EXP phase (two-tailed Welch t test, p < 0.05; Fig. 1B).

In contrast, during REG-phase, *P. putida-*ICE*clc* expressed 748 genes statistically significantly differently compared to *P. putida* without ICE*clc* (Fig. 1A, Data S1). Only 51 of those overlapped with the previous set of genes observed in EXP phase (Fig. 1C, same direction of expression), indicating that more chromosomal genes respond to the presence of ICE*clc* in REG than EXP phase. In REG phase *P. putida-*ICE*clc*, five KEGG pathways were higher expressed, including ribosome synthesis (ko03010), fatty acid metabolism (ko01212), protein export (ko03060) and secretion system (ko03070), while nine pathways were repressed, such as cell cycle (ko04112) and amino acid metabolism (ko00260, ko00330) (Fig. 1B). None of the 14 KEGG pathways differentially expressed in REG phase overlapped with the five pathways higher expressed in EXP phase ICE*clc*-carrying cells.

Most of the genes in the ICE-variable region (coordinates 5,000–50,000), which in case of ICE*clc* contains the *clc* genes, were indistinguishably expressed between EXP and REG–phase cells, concluded from normalized read counts of the ICE*clc* region in *P. putida* 2737 among EXP and REG phase (Fig. S2). On the other hand, the *intB13* gene and genes in the ICE core region (coordinates 1–5,000 and 50,000–100,000) involved in horizontal transmission were more highly expressed in REG than EXP phase (Fig. S2), confirming ICE activation in REG-culture conditions.

### ICE*clc* activates acryptic prophage

**Figure 2:**
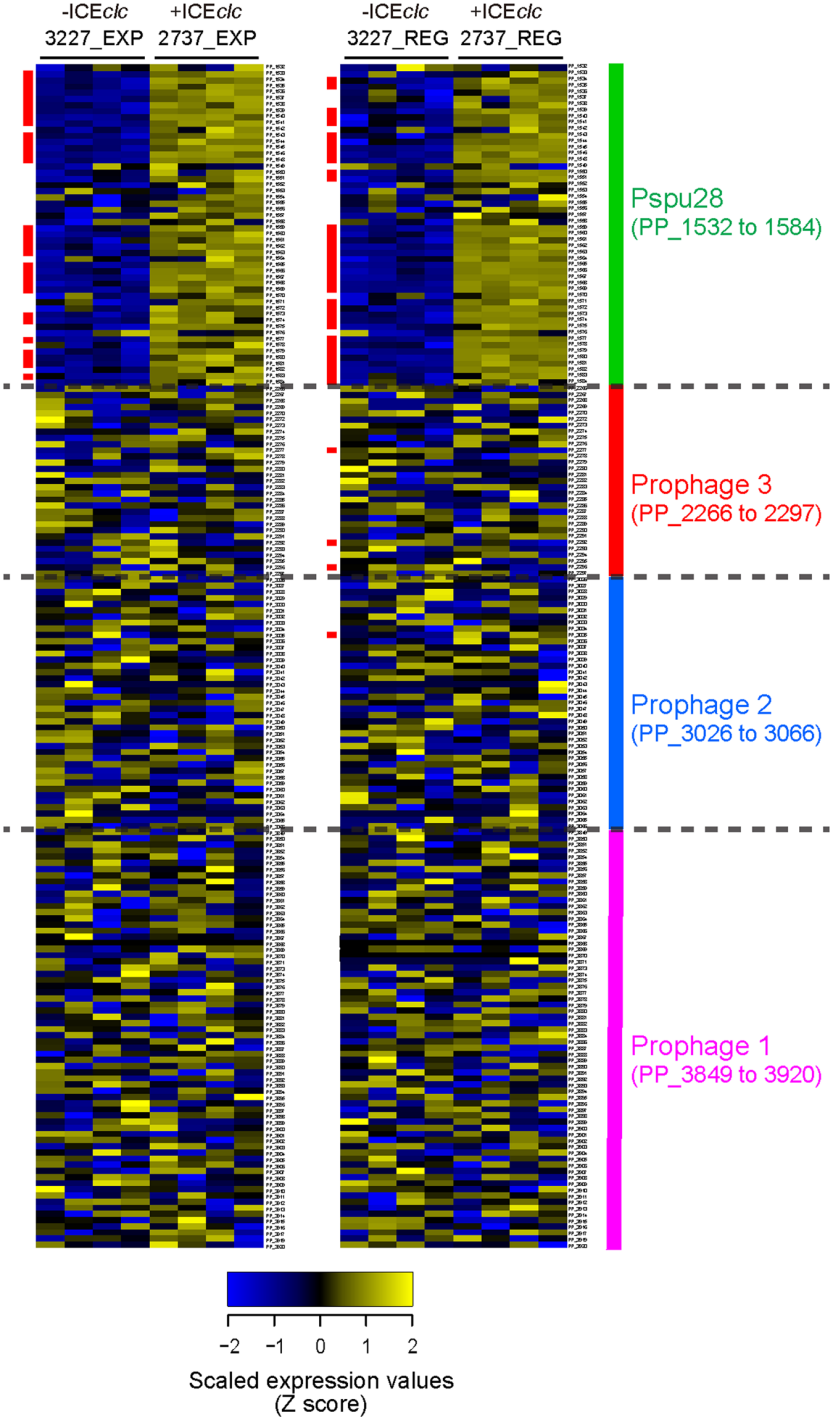
Differential gene expression profiles in four prophage regions of *P. putida* in presence or absence of ICE*clc*. Blue/yellow blocks indicate chromosomal genes (rows) that are up/down regulated in quadruplicate samples (columns) of the indicated *P. putida* strains and growth phases (top). Genes are clustered according to the four prophage regions as indicated on the right. Vertical red lines on the left side of rows indicate genes with a false-discovery rate q-value < 0.01 on each pairwise comparison.

One of the striking unexpected findings in the transcriptome comparisons pointed to a link between ICE*clc* and expression of one out of four prophages in *P. putida* (Fig. 2), which has previously been designated Pspu28^33,34^. Expression of the Pspu28 prophage genes (at position ~1.8 Mb in the genome, Fig. 1) is clearly higher in *P. putida*-ICE*clc* (strain 2737) than in *P. putida* without (strain 3227), and occurs both in EXP and REG phase (Fig. 2). Gene expression in the other prophage regions in the *P. putida* genome was not different in presence or absence of ICE*clc* (Fig. 2).

**Figure 3:**
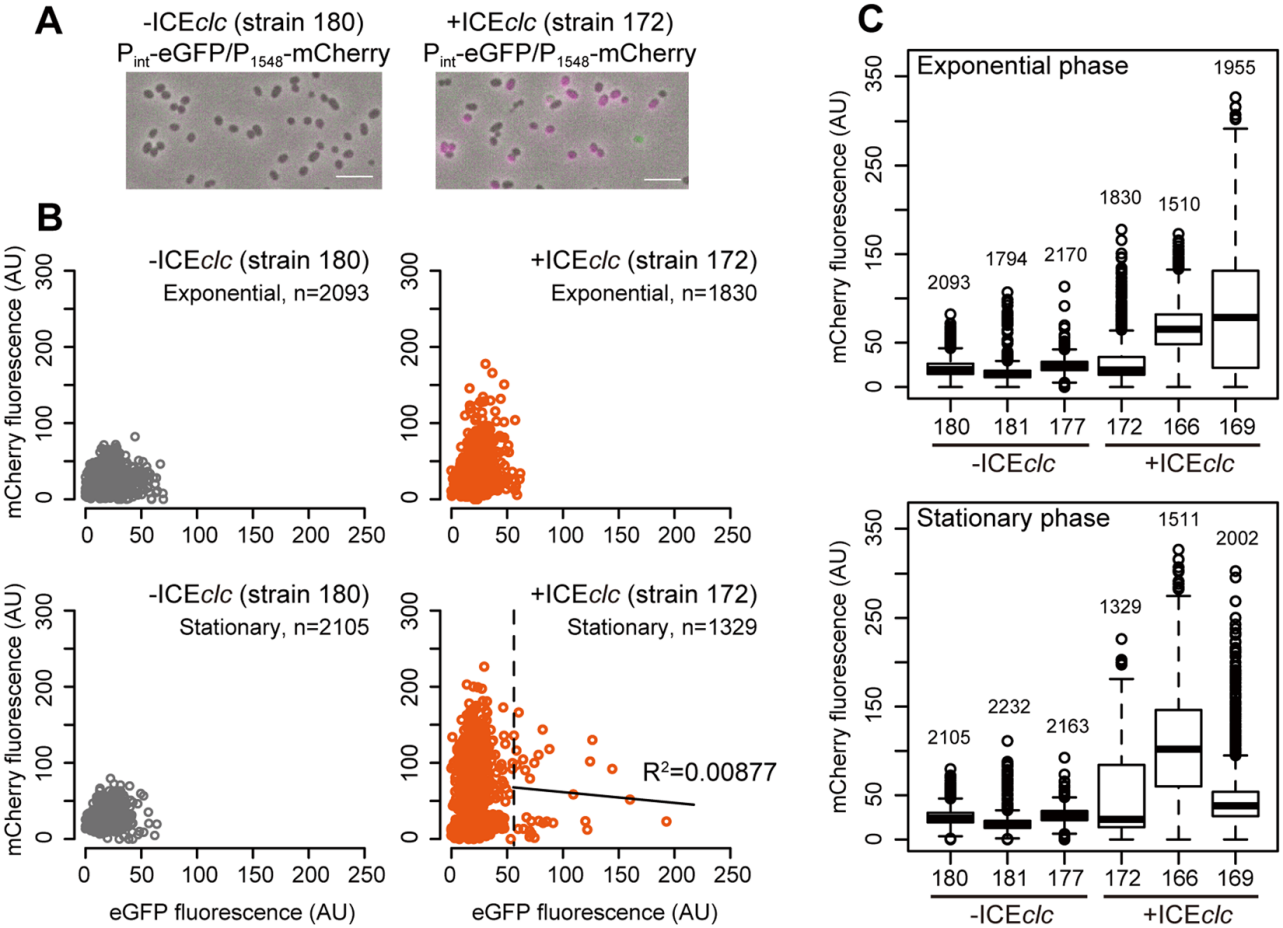
Differential expression of the Pspu28 phage P_1548_ promoter at single-cell level in *P. putida* with or without ICE*clc*. (**A**) Representative micrographs of *P. putida* strains carrying single-copy chromosomal inserts of the P_1548_-*mcherry* and the ICE*clc* bistable P_int_–promoter –*egfp* fusions, via mini-transposon delivery. Images show merged phase–contrast (grey), eGFP (green), and mCherry (magenta). Scale bar indicates 5 μm. (**B**) Scatter plots showing mCherry (from P_1548_) and eGFP (from P_int_) fluorescence intensities among individual cells (circles) of *P. putida* without (strain 180) and with ICE*clc* (strain 172) at exponential and late stationary phases in MM with 3CBA. Threshold (dashed line) between P_int_-active and -inactive cells of strain 172 at late stationary phase was calculated according to Reinhard and van der Meer^71^. Note that mCherry and eGFP fluorescence among the P_int_-active cells is not correlated significantly (R^2^ = 0.00877). (**C**) Box plots showing mCherry (from P_1548_) fluorescence intensity among individual cells of *P. putida* without (strain 180, 181, and 177) and with ICE*clc* (strain 172, 166, and 169) at exponential and late stationary phases in MM with 3CBA. The number of measured cells is indicated for every sample. Note that the different *P. putida* strains have randomly inserted fluorescence reporter constructs.

To confirm that phage genes are expressed in *P. putida*-ICE*clc* cells, we developed dual fluorescence *P. putida* reporter strains carrying single copy insertions of an *mcherry* gene fused with the promoter of PP_1548 (P_1548_-*mcherry*), encoding a Pspu28 phage-specific protein. Expression of PP_1548 was easily noticeable in the global transcriptome and therefore deployed as marker here. The reporter strain further contained an *egfp* gene fused with the ICE*clc* integrase promoter (P_int_-*egfp*), which becomes active in 3–5% of stationary/REG phase transfer-competent cells where ICE*clc* horizontal transmission occurs^28,35^ (Fig. 3A). In agreement with the transcriptome studies, expression levels of P_1548_-*mcherry* were higher in *P. putida*-ICE*clc* than in *P. putida* without ICE*clc* in both EXP and stationary phases on 3CBA (Fig. 3BC). However, the mCherry intensities of single cells among three clones were varying depending on the insertion position of the P_1548_-*mcherry* fusion, some of which were not normally distributed (Fig. S3), suggesting that prophage activation occurs in a sub-population. No correlation was observed between P_1548_-*mcherry* and P_int_-*egfp* expression at single cell level (R^2^ < 0.01, Fig. 3B), indicating the phage is indeed activated by the presence of ICE*clc*, but not specifically induced in the subset of cells that activate P_int_-*egfp*. On the other hand, no obvious differences were observed between *P. putida* with and without ICE*clc* growing on LB; regardless of the ICE presence, P_1548_-*mcherry* was silent in EXP phase but was expressed in a certain fraction of cells in stationary phase (Fig. S4).

### ICE*clc* influences cell motility depending on growth condition

**Figure 4:**
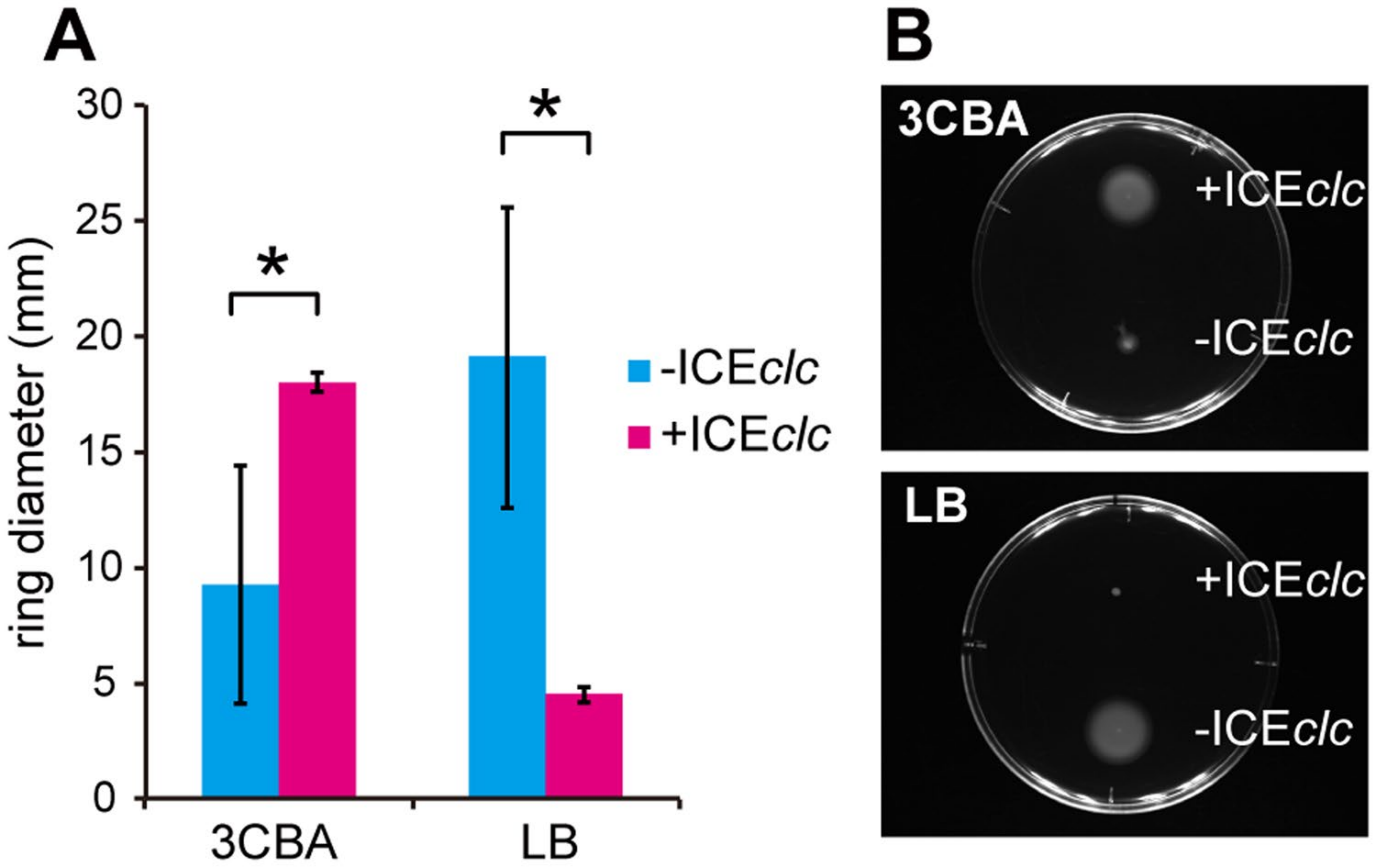
Swimming motility of *P. putida* in the presence/absence of ICE. *P. putida* strains with (strain 2737) and without ICE*clc* (strain 3227) were pregrown in MM with 3CBA or LB and tested on swimming plates (PG medium solidified with 0.3% agar). (**A**) Measurement of the mean colony ring diameter (±1 *SD*, n = 5) after 19 h incubation. Asterisks above bar diagrams indicate significance of difference (P < 0.01) in one-tailed t tests. (**B**) Representative images of different colony sizes on swimming plates after 19 h of incubation of *P. putida* strains precultured in MM with 3CBA (upper panel) or in LB (lower panel).

A second surprising finding from transcriptome comparisons was the higher expression of the *P. putida* flagellar assembly pathway in ICE*clc–*containing strains in EXP phase (Fig. 1B). *P. putida*-ICE*clc* was indeed more motile on agar media than *P. putida* without ICE*clc*, but only when pregrown in minimal medium with 3CBA as carbon substrate (Fig. 4). In contrast, motility after growth on complex medium (LB) was reduced in *P. putida*-ICE*clc* (Fig. 4). These results indicate that ICE*clc* increases the host cell’s motility in the presence of substrate that it can metabolize thanks to the *clc* genes on the ICE.

### Host-induced fitness effects by ICE*clc* are medium dependent

**Figure 5:**
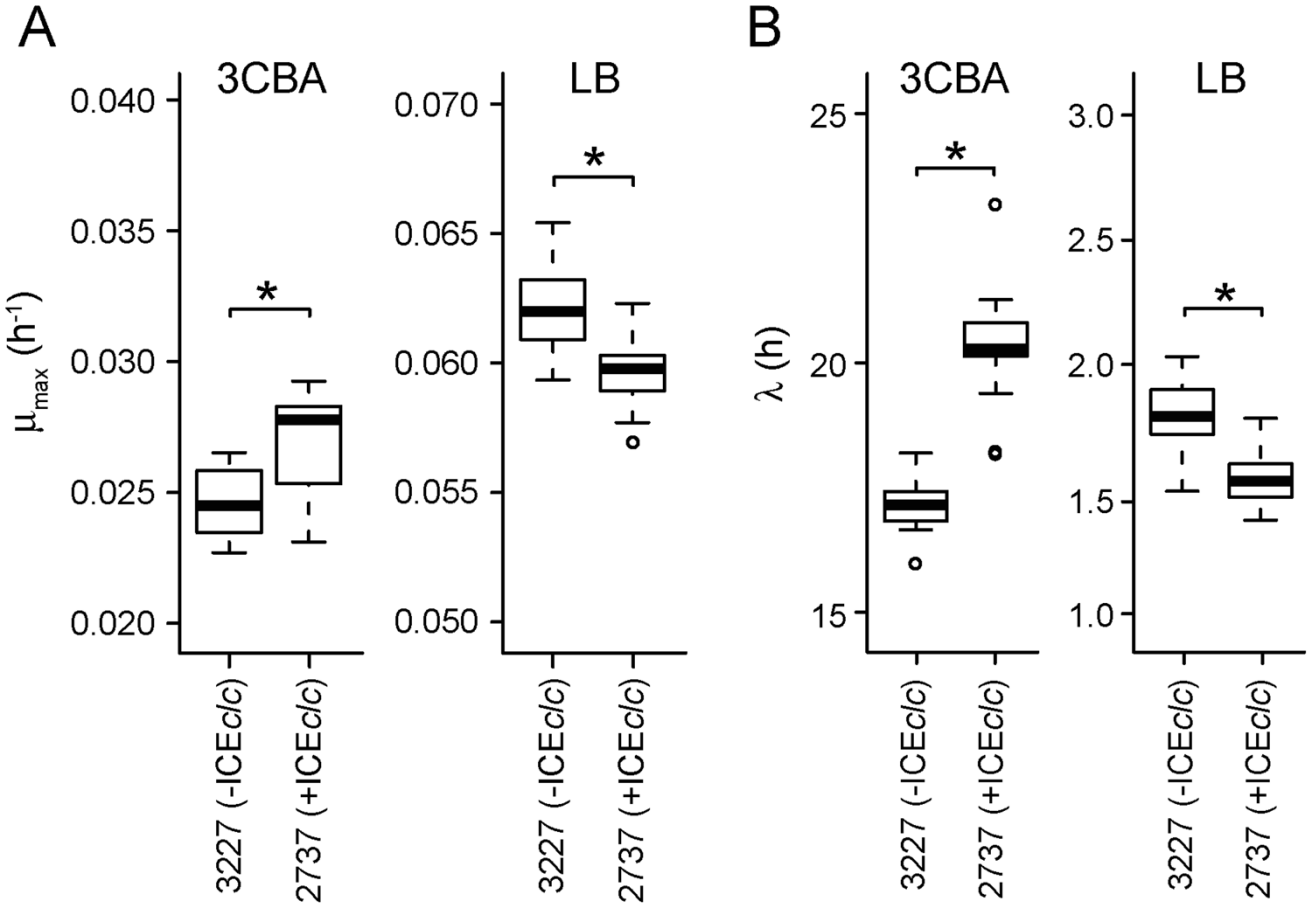
Effect of ICE*clc* on *P. putida* growth fitness. (**A**) Maximum growth rate (μ_max,_ h^-1^) and (**B**) length of lag phase ($\lambda ,$ h), calculated from 20 replicates of each *P. putida* strain growing in MM with 3CBA (left) or in LB (right). Asterisks above box plots indicate significance of difference on each pair (P < 0.0001) in t test.

In order to determine whether ICE*clc* globally impacts fitness of *P. putida*, we compared growth rates of *P. putida* with (strain 2737) and without ICE*clc* (strain 3227) on minimal medium with 3CBA or on complex (LB) medium. The average maximum growth rate (μ_max_) of *P. putida* with ICE*clc* on 3CBA was higher than that without (P < 0.01, Fig. 5A). Given that no difference among the strains was observed in gene expression levels directly involved in 3CBA metabolism, such as the *ben* and *clc* genes (Table S2), the beneficial effect of the ICE on *P. putida* growth could be exerted by other mechanisms. In contrast, the µ_max_ of *P. putida-*ICE*clc* was lower than that of *P. putida* in LB medium (Fig. 5A), indicating that the positive effect of ICE*clc* on growth rate is carbon source (or medium)-dependent. A longer lag phase on 3CBA was observed in *P. putida*-ICE*clc* than *P. putida* without ICE*clc* (Figs 5B, S4). This could be the result of growth arrest of 3–5% cells that had activated ICE*clc* horizontal transmission in the preculture, as previously simulated by modeling^31^.

## Discussion

ICEs are widely present self-transferable elements in bacterial lineages, suggesting adaptive benefits from their presence. However, the global effects of their presence on the host cellular systems and expression networks have been poorly characterized. Our results indicate that, globally, the presence of ICE*clc* improves *P. putida* fitness on the carbon substrate 3CBA, which is exclusively metabolizable thanks to the ICE, but impairs fitness on other carbon substrates.

A previous study using micro-array analysis showed very few differences in host gene expression and less than 1% fitness costs of ICE*clc* on *P. aeruginosa* PAO1 growing on succinate as a non-selective neutral carbon source^30^. The absence of immediate detrimental effects to the host PAO1 led us to conclude that ICE*clc* is a ‘perfect partner leaving very little imprint but with specific selective benefits when needed’^30^, which might be one of the reasons ICEs of this type are widely distributed among bacterial genomes. However, that study could not test the effect of growing ICE*clc–*containing hosts on 3CBA, which is the carbon substrate that leads to the highest activation of the ICE in stationary phase (3–5% of cells) and stimulates its excision and horizontal transmission in such cells (REG conditions)^29^. To complement the impact study of ICE*clc* in PAO1, therefore, we here used a *P. putida* host and ensured that both host without and with ICE*clc* can grow on 3CBA. This was accomplished by introducing the *clc* genes by single copy Tn7 chromosomal delivery into *P. putida* (strain 3227). The growth of this strain is very nearly the same as *P. putida-*ICE*clc* (strain 2737, see below and Fig. S5).

In cells growing exponentially on 3CBA, expression of 2.8% of chromosomal genes (161 of 5671 total genes) was affected in *P. putida* with compared to *P. putida* without the ICE, encompassing five identifiable biological KEGG pathways. In comparison, we previously detected a single expression difference (1 out of 5,900 genes and intergenic regions) between exponentially growing cells on succinate of *P. aeruginosa* PAO1 with and without ICE*clc*^30^. No global pathways of *P. putida* significantly decreased expression in exponentially growing cells in presence of ICE*clc*.

In contrast, in REG phase, 13% of *P. putida* host genes (748 in total) encompassing 14 biological pathways changed expression levels compared to *P. putida* without ICE. Notably, protein export (ko03060), secretion system (ko03070), and ribosome synthesis (ko03010) were activated, while cell cycle (ko04112) and several metabolic pathways (e.g. ko00500, ko00260, ko00330) were repressed. In *P. aeruginosa* PAO1, the expression differences among strains with or without ICE*clc* were also more substantial in stationary than exponential phase on succinate (42 genes significantly changed, out of 5671)^30^. However, there are only two orthologous genes overlapping between those differentially expressed in *P. putida* and *P. aeruginosa* (PP_0356/PA0482, malate synthase G; PP_2351/PA3568, probable acetyl-CoA synthase), suggesting that global changes induced by ICE*clc* are substantially different between those hosts and conditions. The stronger effect of ICE*clc* in 3CBA REG conditions in *P. putida* may be due to the activation of the ICE horizontal transmission program, and might explain the observed reduction in expression of the cell cycle and increased secretion/export systems. Alternatively, there might be specific links between other ICE*clc–*encoded factors, dependent on the host network that we currently do not understand. The difference in the extent of host global gene expression by ICE*clc* between *P. putida* and *P. aeruginosa* PAO1 as hosts is striking. This may partly be an effect of the host, but may also be an exaggerated effect from the carbon substrate 3CBA as opposed to succinate that results in more active ICE*clc* expression^36^.

One of the incidental findings in this study was the observed activation of the cryptic prophage Pspu28 in *P. putida* by the presence of ICE*clc*. Although *P. putida* UWC1 harbors a total of four prophages, only Pspu28 responded to ICE*clc*, suggesting specific crosstalk between them. To our knowledge, this is the first report to show an ICE–phage global interaction. Pspu28 has a size of 40-kb and encodes a filamentous bacteriophage with a capsid-like hexagonal structure, whose relatives are found among *Pseudomonas* and *Shewanella* genomes^37^. The phage can switch between lysogenic and lytic states, but lacks a lytic enzyme (i.e. endolysin), suggesting that it is incapable of actively killing the host. It releases progeny phage particles when the host eventually dies. A previous study reported that Pspu28 phage lysogeny leads to fitness loss of *P. putida* in the rhizosphere through an unknown mechanism^34^. Activation of the Pspu28 phage P_1548_-promoter was heterogeneous among *P. putida* cells carrying ICE*clc* (Fig. S3), but not exclusive to the subpopulation of cells expressing the ICE*clc* P_int_-promoter (Fig. 3). This indicates that the prophage activation is a more general effect of ICE*clc* presence and not linked to the actual ICE horizontal transmission process from a subset of cells. We currently ignore the exact molecular links between ICE and phage activation. Interestingly, Pspu28 activation has previously also been observed as a side-effect of introduction of the conjugative plasmid pCAR1 into *P. putida*^38^. This suggests that the phage is somehow particularly prone for being activated by newly incoming mobile elements, but the ecological reasons or host consequences for this activation are unclear.

Another obvious phenotypic change invoked by the presence of ICE*clc* was swimming motility. *P. putida*-ICE*clc* cells grown on 3CBA more highly expressed genes for flagellar assembly than *P. putida* without ICE (but with the *clc* genes for 3CBA metabolism), whereas such cells also displayed stronger motility. In contrast, *P. putida-*ICE*clc* cultures pregrown on LB specifically repressed motility. This indicates that ICE*clc* controls host motility depending on the carbon source. The finding that *P. putida-*ICE*clc* cells grown on 3CBA move faster is very suggestive for chemotaxis toward the compound, which could be a selective advantage of carrying the ICE to the host metabolizing 3CBA. Although bacterial chemotaxis toward aromatic compounds has been well characterized in various species^39^, specific chemotaxis to 3CBA has not been described. 3CBA chemotaxis may be mediated by the *orf2848* gene product encoded by ICE*clc*, which shares the same functional domains throughout the entire protein sequence with PcaK, a bifunctional membrane protein required for both transport of and chemotaxis to 4-hydroxybenzoate^40^.

In terms of overall fitness, the introduction of ICE*clc* into *P. putida* thus caused mixed effects. Despite the differential expression of 2.8% of genes in the *P. putida* genome in presence of ICE*clc* in exponentially growing cells, and despite activation of the Pspu28 prophage, the growth rate on 3CBA of *P. putida-*ICE*clc* was actually slightly more elevated than that of *P. putida* 3227 (without ICE, but with the *clc* genes, Fig. 5A). The lag phase of *P. putida-*ICE*clc* on 3CBA, however, is longer than that of *P. putida* 3227 (Fig. 5B), which is likely due to the limited growth of the 3–5% subpopulation of stationary phase cells that activate ICE*clc* horizontal transmission and perish upon nutrient restimulation, as previously attested by single-cell microscopy^28,29^. The poor growth of ICE*clc-*transmitting cells may thus also be responsible for the observed reduction in REG–phase expression of biological pathways, such as cell cycle (ko04112) and amino acids (ko00260 and ko00330) or carbohydrate (ko00630 and ko00500) metabolism. On average, therefore, ICE*clc* increases population fitness on 3CBA as carbon substrate (i.e., higher growth rate, increased chemotaxis), while its fitness impacts (i.e., longer lag phase) are minimized by restricting horizontal transmission to a small proportion of cells in the population^29^. In contrast, growth rates on LB were slightly lower (Fig. 5), without any obvious Pspu28 phage activation (Fig. S4). Although we did not specifically measure global transcriptome changes in LB-grown cells, growth rate differences suggest that ICE*clc* is imposing a slight fitness costs under such conditions in *P. putida*. Fitness differences, globally speaking, do not seem to be influenced by the induction or not of Pspu28 phage genes.

Overall, host-mobile element partnerships seem to have evolved multiple times conceptually similar systems to optimize the balance between host-incurred costs and benefits^41,42^. ICE*clc* core gene expression remains silent in exponentially-growing cells, which is the result of global repression by the TetR-type MfsR transcription regulator^31^. In stationary phase, ICE*clc* horizontal transmission is restricted to a subpopulation of cells as a result of a bistable switch in a hitherto uncharacterized mechanism^34^. Similarly, but mechanistically different to ICE*clc*, temperate phages and other ICEs limit horizontal transmission by deploying a strong double negative feedback circuit to repress their lytic cycle^21,25,43,44^. Conjugative plasmids are known to exploit specific H-NS-like stealth proteins, temporarily silencing their gene expression upon entry into a new recipient cell in order to reduce fitness impairment^17–19^.

Our study thus indirectly shows the large impact on host cells of ICE*clc* during its active transmission phase (i.e., REG phase cells). At this stage we can only infer this impact indirectly because of the masking effect of the large proportion of cells in REG phase in which ICE*clc* remains silent compared to those in which it is active (3–5%). Whereas a number of studies have revealed host genome-wide responses during lytic phage infection^45–51^, the differences during vertical (lysogenic cycle) and horizontal transmission stages of temperate phages (lytic cycle) have largely remained unexplored^20,52,53^. Similarly, specific host transcriptome changes during plasmid horizontal transmission have been poorly documented^38,54–56^. It will be interesting to characterize the host-ICE*clc* partnership further, in particular in cells actively undergoing ICE horizontal transmission, but this will have to be addressed by separating individual subpopulations.

## Methods

### Bacterial strains and culture media

*Escherichia coli* DH5α (Gibco Life Technologies) and DH5αλ*pir*^31^ were routinely grown at 37 °C on Luria-Bertani (LB) medium^57^ for plasmid constructions. Derivatives of *P. putida* UWC1, a spontaneous rifampicin resistant strain of KT2440, used in this study (Table S3) were cultured at 30 °C on LB or type 21 C minimal medium (MM)^58^ containing 5 mM 3CBA. If necessary, antibiotics were added at the following concentrations; kanamycin 25 μg mL^−1^, gentamicin 20 μg mL^−1^, ampicillin 100 μg mL^−1^, and tetracycline 50 μg mL^−1^.

### RNA-sequencing using bulk populations

*P. putida* strains were precultured in MM containing 5 mM 3CBA until exponential growth phase (OD = ~0.6) and transferred into fresh medium with 100-fold dilution. The cells of four replicate cultures were collected either at exponential phase (OD = ~0.6) or from late-stationary phase cells grown for 96 h on 3CBA, and then further stimulated for 4.5 h with additional 5 mM 3CBA (REG phase). Collected cells were immediately treated with RNAlater (Ambion). Total RNA was extracted using the hot phenol method^59^, and the residual co-extracted genomic DNA was digested by incubation with Turbo DNase (Life Technologies) following manufacturer’s instructions. The RNA was further purified using the RNeasy MiniElute cleanup kit (Qiagen), and rRNA was specifically depleted using Ribo-Zero rRNA removal kit Bacteria (Epicentre). The quality and quantity of RNA were measured using a Fragment Analyzer (Advanced Analytical) and a High Sensitivity RNA Analysis Kit (Advanced Analytical). cDNA libraries were generated using a ScriptSeq Complete Kit Bacteria (Epicentre) following strand-specific library preparation protocol. The indexed cDNA libraries were pooled and sequenced on Illumina HiSeq. 2000 platform with 101-nt single-end reads.

### Bioinformatics

High quality reads (mean quality score >35) were mapped to the reference sequences of *P. putida* strain KT2440 chromosome (refseq NC_002947) and ICE*clc* (Genbank accession AJ617740) using Bowtie allowing 2 bp mismatches for a 101 bp read^60^. Reads mapped to a unique position were used for subsequent analysis, removing a massive amount of reads from rRNA genes. Mapped data were converted to the BAM format using Samtools^61^, then read counts for coding sequences (CDSs) were obtained using HTseq^62^. Coverage was obtained using BEDtools^63^. We obtained 2.1 to 10 million reads mapped to CDSs per sample.

Differential expression analysis was performed using the TMM normalization-edgeR exact-test iteration protocol of the Bioconductor package TCC^64^. Multidimensional scaling analysis was performed using the plotMDS function in edgeR with default arguments. A false discovery rate (q.value) of <0.05 was considered statistically significantly differentially expressed. Pathway level activity analysis^32^ was conducted to detect gene set level expression changes. KEGG orthology ID was assigned to chromosomally encoded genes using blastKOARA of KEGG^65^, and subsequently a total of 83 pathways, each of which contained more than 10 genes assigned were tested. In this method, pathway activity is essentially the sum of Z-score of gene expression values of gene set members whose expression values were shifted to the major direction. Read counts were normalized across samples using internal functions of TCC package, then used as input data for the Z-score analysis option of Bioconductor package GSVA^66^.

### Fitness assay

*P. putida* strain 3227 (-ICE*clc*) and 2737 (+ICE*clc*) were precultured in MM containing 5 mM 3CBA for 72 h until stationary phase (OD = 1.5), and transferred with 100-fold dilution into fresh LB or MM containing 2 mM 3CBA. The optical densities (OD_600_) of fresh cultures with 20 replicates were monitored at intervals of 10 min for 48 h, using Tecan Spark 10 M microplate reader. Maximum growth rate (μ_max_) and length of lag-phase were calculated using the R package grofit^67^ with smooth.gc parameter 0.5, in which we used logistic model fitting for LB and smoothing spline method with 100 bootstrapping runs for MM containing 3CBA cultures.

### Single-cell gene expression assay

A 334-bp promoter region of the PP_1548 gene was amplified with primers 160701 (5′-tttACTAGTCCGAAAGCTCCTACACACTG) and 160702 (5′-tttGGTACCATGGGGTGTTGCTCCGTG) using genomic DNA of *P. putida* UWC1 as a template, and digested with SpeI and KpnI. An *mcherry* gene fragment was amplified using primers 160614 (5′-tttGGTACCTTAACTTTTAAGGAGGAAAAACATA) and 160615 (5′-TTATTTGTACAGCTCATCCATG) plus pCK218-Pint-gfp-PinR-mcherry as a template^35^, and digested with KpnI. The two digested fragments were cloned into SpeI and DraI sites of the mini-Tn5 vector pTn*Mod*-OTc. The resulting plasmid pTn*Mod*-P1548-mcherry and another mini-Tn5 plasmid pCK218-jim1 containing P_int_-*egfp*^68^ were both introduced into either *P. putida* strain 3227 or 2737 by electroporation. Three individual clones of each transformant containing the dual reporter genes were cultured in MM plus 5 mM 3CBA or LB and collected at exponential and late stationary phases to use for subsequent microscopic assays. Gene expression analysis at single cell level was performed as previously described^69^, using a Zeiss AxioObserver.Z1 inverted microscope, equipped with either Zeiss AxioCam MRm or AxioCam 503mono CCD, and 100 x /1.4 oil immersion Plan-Apochromat lens at exposure times of 350 ms for phase contrast and 100 ms for fluorescence images. The light source used for fluorescence imaging was Zeiss Colibri.2. Filters used for eGFP and mCherry were Zeiss 38HE and Semrock mCherry-B-000, respectively.

### Motility assay

Bacterial cells were pregrown in LB for 18 h or in MM supplied with 5 mM 3CBA for 72 h until stationary phase, and 1-µl aliquots of precultures adjusted to a culture turbidity of 0.6 at 600 nm were spotted on PG medium (0.5% Difco Proteose Peptone No.3, 0.2% glucose) solidified with 0.3% agar^70^. After 19 h incubation at 25 °C, diameters of swimming rings were measured (n = 5).

### Data availability

RNA-seq datasets generated during this study are deposited and available in the Sequence Read Archives of National Center for Biotechnology Information (NCBI), European Bioinformatics Institute (EBI) and DNA Data Bank of Japan (DDBJ), http://trace.ddbj.nig.ac.jp/DRASearch, under accession number DRA005831.

## Electronic supplementary material

Supplementary information

Data S1
