# Supplementary material for: Physiological and transcriptome changes induced by Pseudomonas putida acquisition of an integrative and conjugative element
Source: Sci Rep. 2018 Apr 3;8:5550. doi: 10.1038/s41598-018-23858-6 (PMC5882942; doi:10.1038/s41598-018-23858-6)
Supplement: Supplementary file 1 — Supplementary information [file 41598_2018_23858_MOESM1_ESM.pdf]

Supplementary information to

Physiological and transcriptome changes induced by *Pseudomonas putida* acquisition of an integrative and conjugative element

Ryo Miyazaki*, Hirokazu Yano, Vladimir Sentchilo, and Jan Roelof van der Meer

* Corresponding author

Email: ryo.miyazaki@aist.go.jp

Supplementary Figure S1-S5

Supplementary Table S1-S3

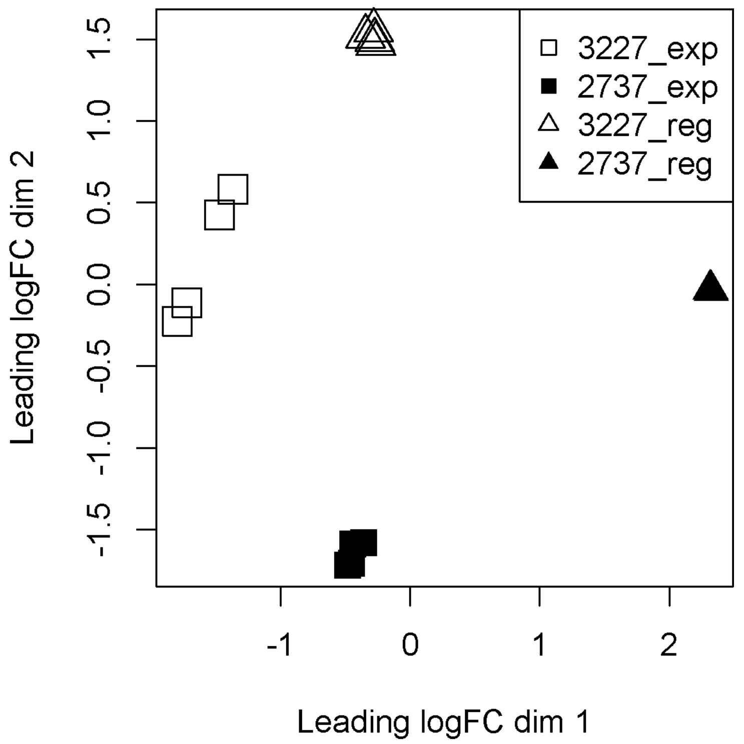

Fig. S1. Multidimensional scaling (MDS) analysis of transcriptomes of strain 2737 and 3227. The distance between all 16 samples is shown by a MDS plot. Only chromosomal genes (i.e., without ICE*clc*) were used for calculations. Replicates of the different experimental treatments are depicted by the same symbols.

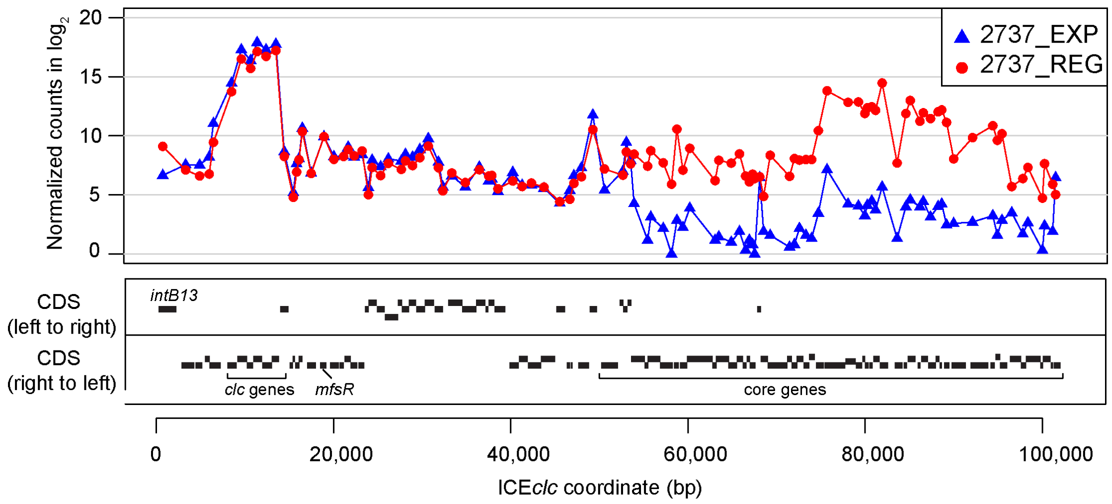

Fig. S2. Comparison of absolute expression levels of ICE*clc* genes. Normalized read counts from whole cultures of *P. putida* carrying ICE*clc* (strain 2737) at exponential (blue triangle) and REG (red circle) were compared. The value for each gene represents a median normalized read count from quadruplicate measurements. Gene organization and coordinates are indicated at the bottom.

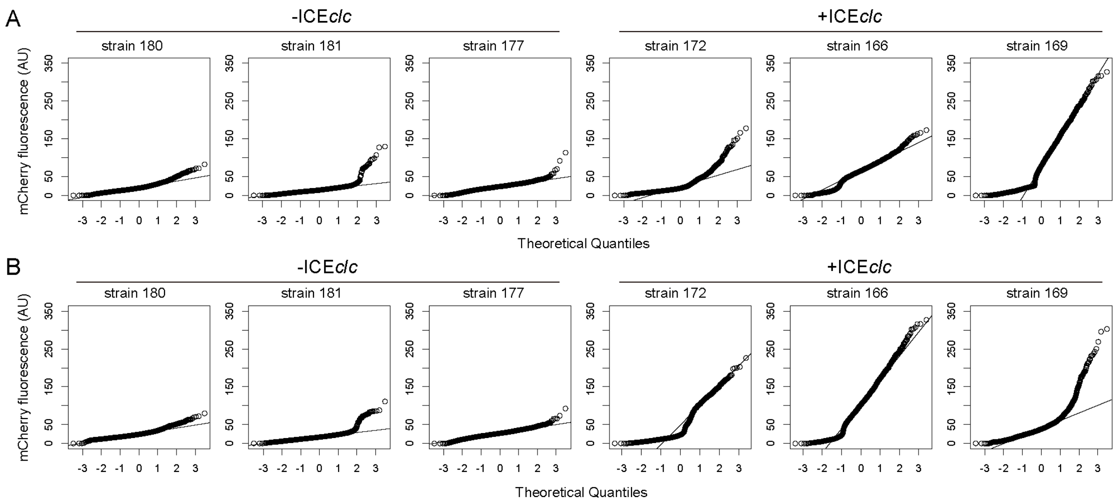

Fig. S3. Distribution of mCherry fluorescence intensities in single cells of *P. putida* with or without ICE*clc*. Normal quantile-quantile plots showing mCherry (from P_{1548}) fluorescence intensities in single cells of *P. putida* strains without (strain 180, 181, and 177) and with ICE*clc* (strain 172, 166, and 169) at (A) exponential and (B) late stationary phases in MM with 3CBA. A straight line in each panel passes through the first and third quartiles, and indicates the normal distribution of the data points. Data points significantly deviating from this line and themselves laying on a line with a different angle are indicative for a second (normal distributed) subpopulation.

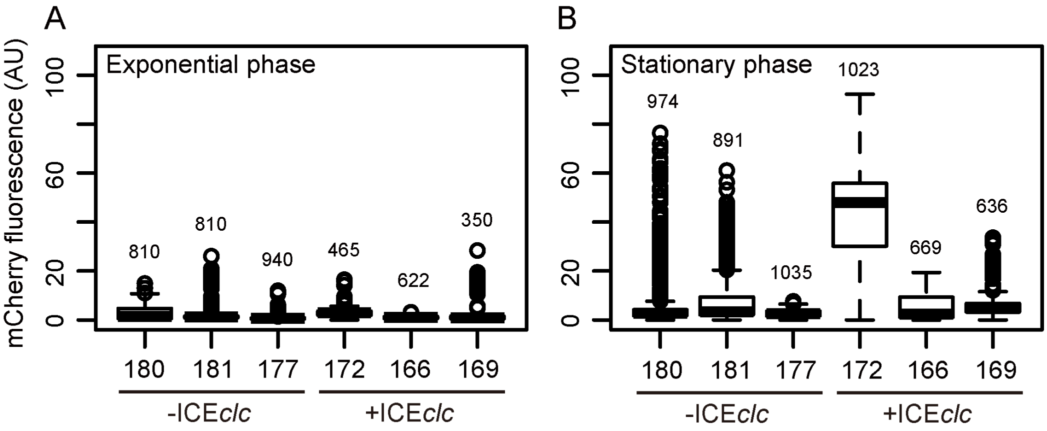

Fig. S4. Differential expression of the Pspu28 phage P_{1548} promoter at single-cell level in *P. putida* with or without ICE*clc* in LB. Box plots showing mCherry fluorescence intensity expressed from a single copy P_{1548}-promoter fusion among individual cells of *P. putida* without (strain 180, 181, and 177) or with ICE*clc* (strain 172, 166, and 169) at (A) exponential and (B) late stationary phases in LB. Number of cells measured is indicated above each box. Note that each strain has the fluorescence reporter construct integrated at different insertion sites on the *P. putida* genome.

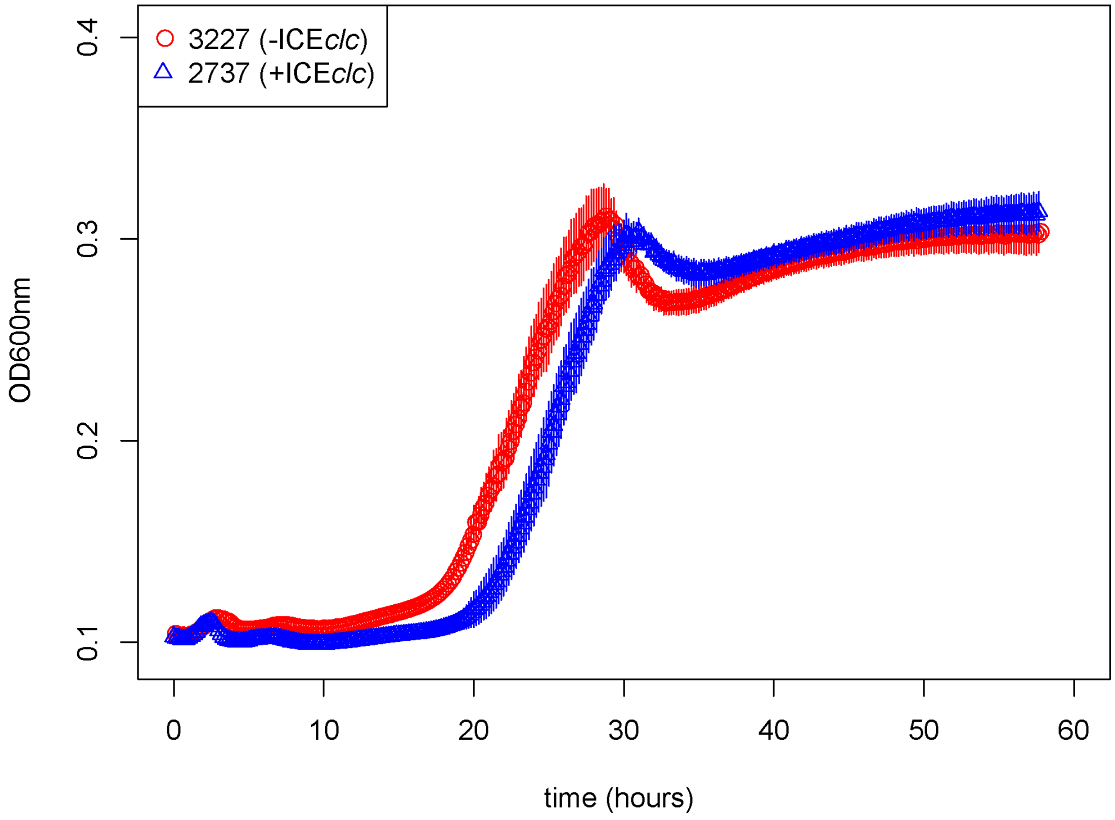

Fig. S5. Growth of *P. putida* with or without ICE*clc* in minimal medium with 3CBA. Data points show mean absorbance (red circle for strain 3227, blue triangle for strain 2737) and standard deviation (colored vertical lines) at each time point, calculated from 20 replicates.

Table S1. Data statistics of RNA-seq

Sample name[a]	Replicate	Description	# total read	# read uniquely mapped to CDS
2737_exp	1	wild-type ICE*clc*	15,337,994	7,292,776
2737_exp	2	wild-type ICE*clc*	19,129,610	8,861,266
2737_exp	3	wild-type ICE*clc*	17,106,374	8,245,541
2737_exp	4	wild-type ICE*clc*	20,415,323	10,175,137
3227_exp	1	*clc* operon, without ICE*clc*	19,069,446	9,198,360
3227_exp	2	*clc* operon, without ICE*clc*	19,723,992	9,283,587
3227_exp	3	*clc* operon, without ICE*clc*	16,726,686	7,359,986
3227_exp	4	*clc* operon, without ICE*clc*	20,055,437	9,354,381
2737_reg	1	wild-type ICE*clc*	15,771,213	5,791,919
2737_reg	2	wild-type ICE*clc*	16,880,241	6,085,459
2737_reg	3	wild-type ICE*clc*	18,228,346	6,106,533
2737_reg	4	wild-type ICE*clc*	18,393,521	6,631,124
3227_reg	1	*clc* operon, without ICE*clc*	15,868,258	6,267,153
3227_reg	2	*clc* operon, without ICE*clc*	14,715,933	6,060,455
3227_reg	3	*clc* operon, without ICE*clc*	15,682,202	6,309,843
3227_reg	4	*clc* operon, without ICE*clc*	16,620,203	6,844,388

[a] strain names and culture conditions are indicated.

Table S2. Differential expression of genes directly involved in 3CBA metabolism between *P. putida* with and without ICE*clc*[a]

Locus tag	Gene	m.value	q.value	Product
PP_3161	*benA*	0.129847	0.948547	benzoate 1,2-dioxygenase subunit alpha
PP_3162	*benB*	0.033065	1	benzoate 1,2-dioxygenase subunit beta
PP_3163	*benC*	0.036315	1	benzoate 1,2-dioxygenase electron transfer component
PP_3164	*benD*	0.064043	1	1,6-dihydroxycyclohexa-2,4-diene-1-carboxylate dehydrogenase
CAE92861.1	*clcA*	-0.117217	0.9591984	chlorocatechol 1,2-dioxygenase
CAE92860.1	*clcB*	-0.128854	0.9449524	chloromuconate cycloisomerase
CAE92858.1	*clcD*	0.056392	1	dienelactone hydrolase
CAE92857.1	*clcE*	0.470833	0.0940129	maleylacetate reductase

[a] Data was extracted from Table S2, comparing strain 3227 (without ICE*clc*) and 2737 (with ICE*clc*) transcriptomes at EXP phase.

Table S3. *Pseudomonas putida* UWC1 derivatives used in this study.

Strain number	Description	Characteristics	Reference
2737	*Pseudomonas putida* UWC1-ICE*clc*	*P. putida* UWC1, a spontaneous rifampicin resistance derivative of KT2440, with one copy of ICE*clc*	[1]
3227	*Pseudomonas putida* UWC1-*clc* operon	*P. putida* UWC1 with specific chromosomal insertion of mini-Tn*7*(*clc* operon)	[2]
166, 169, 172	*Pseudomonas putida* UWC1-ICE*clc*, P_{int}-*egfp*, P_{1548}-*mcherry*	Derivative of strain 2737 with random chromosomal insertions of mini-Tn*5*(P_{int}-*egfp*) and mini-Tn*5*(P_{1548}-*mcherry*). Three independent clones, Km^R, Tc^R	This study
177, 180, 181	*Pseudomonas putida* UWC1-*clc* operon, P_{int}-*egfp*, P_{1548}-*mcherry*	Derivative of strain 3227 with random chromosomal insertions of mini-Tn*5*(P_{int}-*egfp*) and mini-Tn*5*(P_{1548}-*mcherry*). Three independent clones, Km^R, Tc^R	This study

References

1. Sentchilo V, Czechowska K, Pradervand N, Minoia M, Miyazaki R, van der Meer JR. Intracellular excision and reintegration dynamics of the ICEclc genomic island of Pseudomonas knackmussii sp. strain B13. Mol Microbiol. 2009;72(5):1293-1306.
2. Reinhard F, Miyazaki R, Pradervand N, van der Meer JR. Cell differentiation to "mating bodies" induced by an integrating and conjugative element in free-living bacteria. Curr Biol. 2013;23(3):255-259.
